# Phenotype profiling of white-nose syndrome pathogen *Pseudogymnoascus destructans* and closely-related *Pseudogymnoascus pannorum* reveals metabolic differences underlying fungal lifestyles

**DOI:** 10.12688/f1000research.15067.2

**Published:** 2018-07-17

**Authors:** Vishnu Chaturvedi, Holland DeFiglio, Sudha Chaturvedi

**Affiliations:** 1 Mycology Laboratory, New York State Department of Health, Albany, NY, 12208, USA; 2 Department of Biomedical Sciences, School of Public Health, University at Albany, Albany, NY, 12208, USA

**Keywords:** Psychrophilic fungi, phenotype microarray, metabolism, catabolism, gene function

## Abstract

**Background:** *Pseudogymnoascus* *destructans*, a psychrophile, causes bat white-nose syndrome (WNS). *Pseudogymnoascus pannorum*, a closely related fungus, causes human and canine diseases rarely. Both pathogens were reported from the same mines and caves in the United States, but only *P. destructans *caused WNS. Earlier genome comparisons revealed that *P. pannorum* contained more deduced proteins with ascribed enzymatic functions than *P. destructans*.

**Methods: **We performed metabolic profiling with Biolog PM microarray plates to confirm *in silico* gene predictions.

**Results:** *P. pannorum* utilized 78 of 190 carbon sources (41%), and 41 of 91 nitrogen compounds (43%) tested. *P. destructans* used 23 carbon compounds (12%) and 23 nitrogen compounds (24%). *P. destructans* exhibited more robust growth on the phosphorous compounds and nutrient supplements (83% and 15%, respectively) compared to *P. pannorum* (27% and 1%, respectively.). *P. pannorum* exhibited higher tolerance to osmolytes, pH extremes, and a variety of chemical compounds than *P. destructans*.

**Conclusions: **An abundance of carbohydrate degradation pathways combined with robust stress tolerance provided clues for the soil distribution of *P. pannorum*. The limited metabolic profile of *P. destructans* was compatible with *in silico* predictions of far fewer proteins and enzymes. *P. destructans* ability to catabolize diverse phosphorous and nutrient supplements might be critical in the colonization and invasion of bat tissues. The present study of 1,047 different metabolic activities provides a framework for future gene-function investigations of the unique biology of the psychrophilic fungi.

## Introduction

*Pseudogymnoascus destructans* causes white-nose syndrome (WNS), a disseminated disease afflicting hibernating bats in North America since 2006 ^1– 3^. WNS is linked to mass mortality and now afflicts bats over large geographic areas in the United States and Canada. *P. destructans*’ pathogenic mechanisms remain mysterious especially as no other human or animal fungal pathogen expresses virulence attributes at such low temperatures. *Pseudogymnoascus pannorum*, a closely related fungus, is widely distributed in the soil and substrates of caves and mines in North America ^3^. *P. pannorum* grows both at psychrophilic and mesophilic temperature ranges and causes human and canine diseases rarely ^4^. However, *P. pannorum* does not cause any disease in hibernating bats. These facts raise the exciting possibilities that *P. destructans* is more specialized for the pathogenic lifestyle on bats while *P. pannorum* successfully colonizes a broader range of substrates in nature.

Environmental studies on the psychrophilic and psychrotolerant fungi documented the versatility of *Pseudogymnoascus* ( *Geomyces*) *pannorum* for the utilization of complex carbohydrates and keratin-enriched substrates, and tolerance to high salt ^5– 7^. Additional laboratory studies demonstrated extensive saprotrophic enzymatic activities that would allow resource capture by the non-pathogenic *Pseudogymnoascus* species vis-a-vis *P. destructans* ^8, 9^. *P. destructans* is known to secrete proteolytic, lipolytic, and keratinolytic exoenzymes, and possesses specialized catabolic activities that contribute to its growth and survival in the nutrient-poor caves and mines ^2, 10^.

Although their draft genomes are similar in size (~30 Mb), there are numerous repeats and far fewer proteins and enzymes in *P. destructans* (2,052 proteins) than in *P. pannorum* (2,734 proteins) ^11^. In the present study, we report the results of extensive Biolog Phenotype Microarray metabolic profiling to confirm *in silico* gene predictions, and find clues for the different lifestyles of these psychrophilic fungi.

## Methods

The metabolic analysis was conducted using *P. destructans* (M1379) and *P. pannorum* (M1372) ^11^. The PM1-10 and PM21, 23–25 phenotype microarray plates were procured from Biolog, Hayward, CA. The fungal spores were harvested in sterile water from 3 - 5-week-old, heavily sporulating culture on potato dextrose agar (PDA) flasks at 15°C. In preliminary experiments, spore counts and viability were determined on agar plates using a hemocytometer and colony forming units (CFU). For the final tests, the spores were harvested, washed once in sterile water by centrifugation, and the suspension adjusted to an OD _600_ = 0.2 (transmittance = 62%). This suspension equated to between 550 and 950 spores per well via hemocytometer count, and 250–500 spores per well by CFU. In preliminary experiments, the two fungi grew at different growth rates and comparable growth was observed after day 7 for *P. pannorum* and day 10 for *P. destructans* (details not shown). Further incubation of the plates beyond the observation period did not change the observed growth pattern.

The PM plates were inoculated per Biolog protocol and incubated at 15°C ^12, 13^. The presence or absence of growth was measured by OD _600_ on day 10 for *P. destructans*, and day 7 for *P. pannorum*. Negative control wells were weakly growth positive for both *P. destructans* and *P. pannorum*. This observation was also reported for Biolog PM plates in another study ^13^. Therefore, the corresponding negative control well reading from each experiment were averaged together and used to normalize the OD values averages for each test compound. For the heat map visualization, the negative control reading was assigned a score of 0.0 and the positive growth scored on a 0.0 – 1.0 scale. The phenotypic assay was repeated once. The limited dataset precluded any quantitative statistical analysis.

## Results

**Figure 1.**
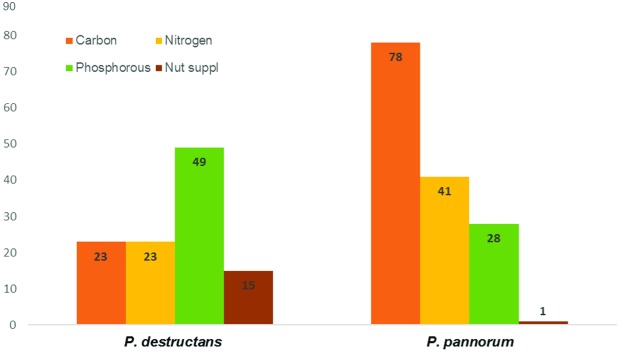
A comparison of carbon, nitrogen, phosphorous and nutrient supplements utilized by *Pseudogymnoascus destructans* and *Pseudogymnoascus pannorum*.

**Figure 2.**
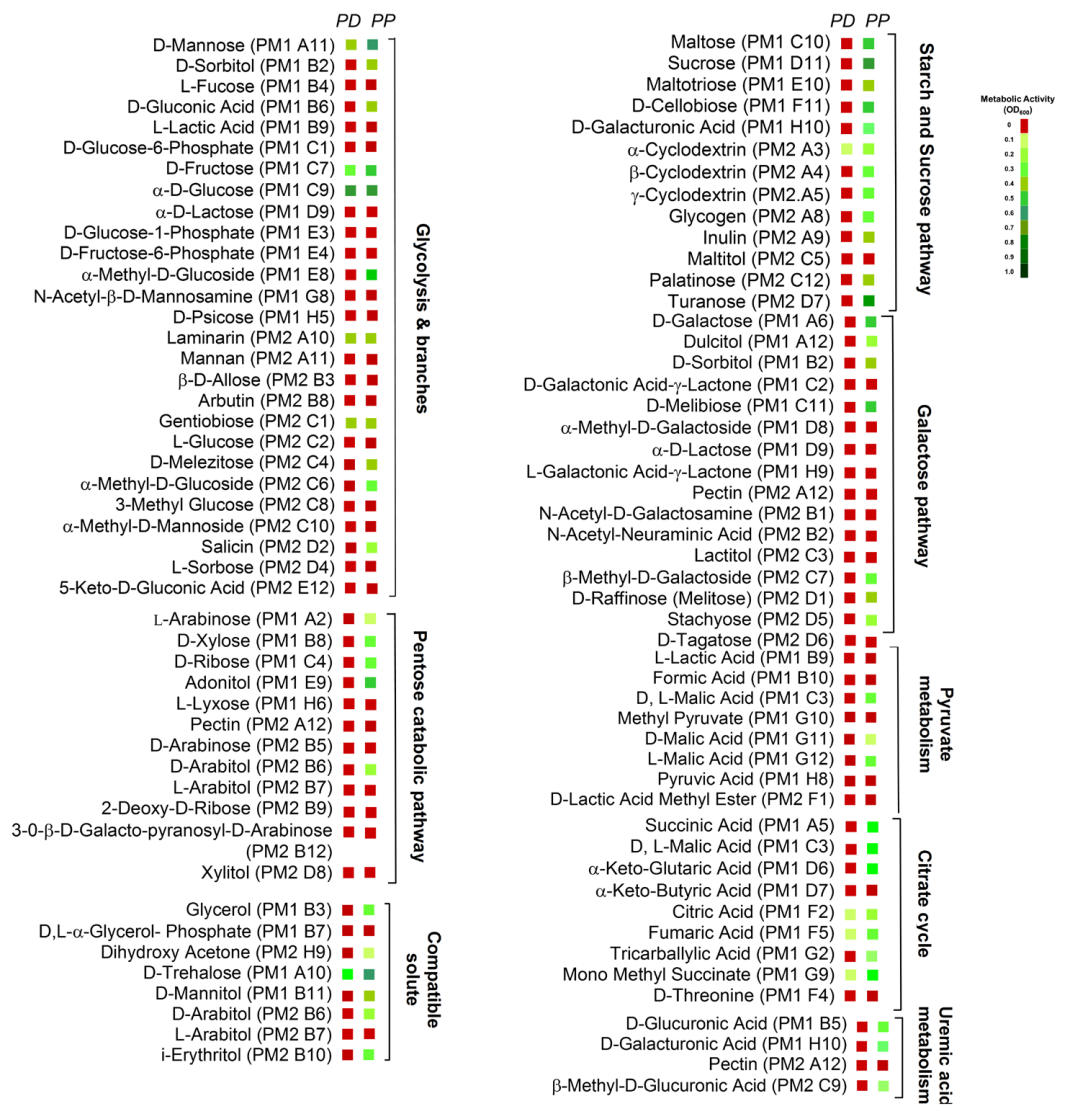
Catabolism of Carbon compounds by *Pseudogymnoascus destructans* (PD) and *Pseudogymnoascus pannorum* (PP). The details of test set-up and end point reading are described in the methods. For the heat map visualization, the negative control reading was assigned a score of 0.0 and positive growth scored on a 0.0 – 1.0 scale.

**Figure 3.**
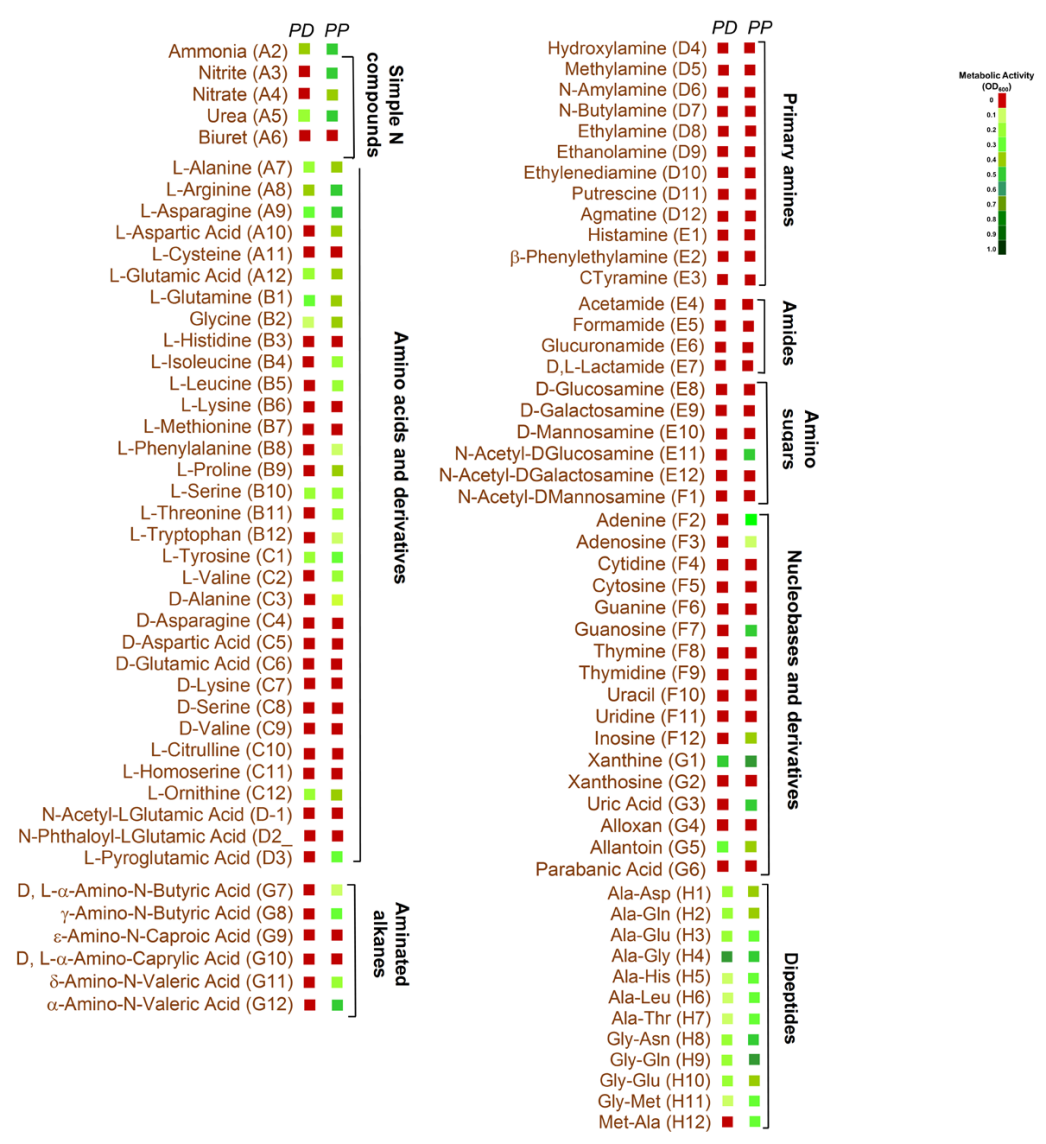
Use of nitrogen compounds by *Pseudogymnoascus destructans* (PD) and *Pseudogymnoascus pannorum* (PP). The details of test set-up and heat map are similar to Figure 2.

**Figure 4.**
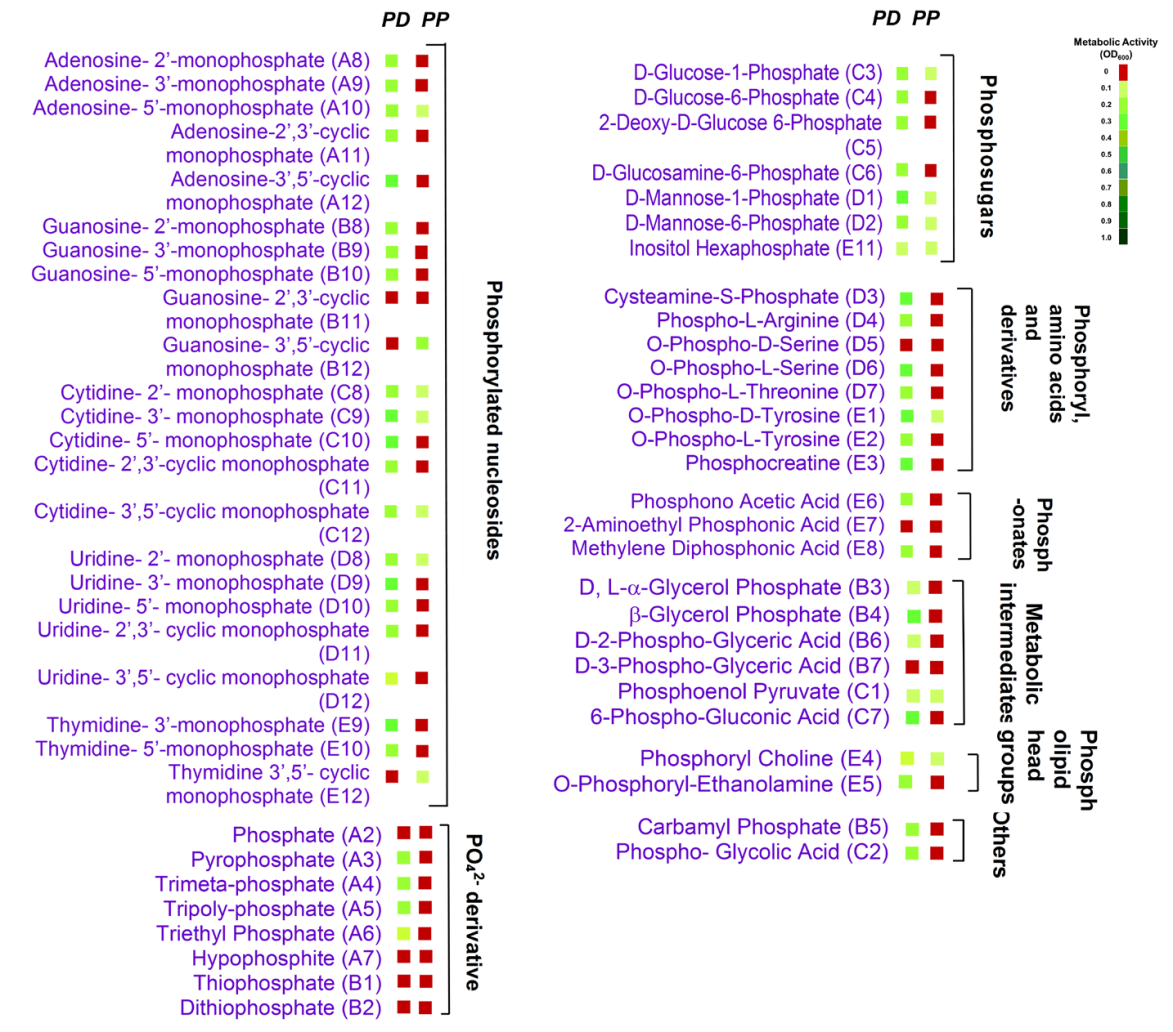
Use of phosphorous compounds by *Pseudogymnoascus destructans* (PD) and *Pseudogymnoascus pannorum* (PP). The details of test set-up and heat map are similar to Figure 2.

**Figure 5.**
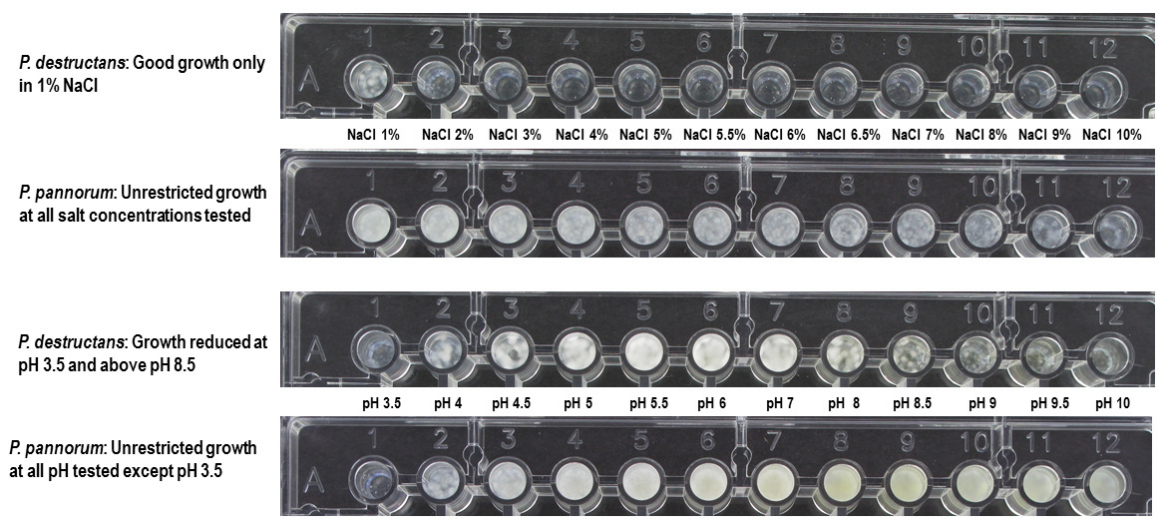
Growth of *Pseudogymnoascus destructans* and *Pseudogymnoascus pannorum* under high salt and pH extremes.

Nearly 1,047 different metabolic activities were analyzed for each test fungus (Datasets 1–4 ^14^). *P. pannorum* metabolized far more carbon and nitrogen compounds; *P. destructans* exhibited prominent activity on phosphorous sources and nutrient supplements ( Figure 1). *P. pannorum* utilized 78 of 190 carbon sources (41%), and 41 of 91 nitrogen sources (43%) tested. *P. destructans* used 23 carbon compounds (12%) and 23 nitrogen compounds (24%). *P. destructans* exhibited more robust growth on the phosphorous sources and nutrient supplements (83% and 15%, respectively) compared to *P. pannorum* (27% and 1%, respectively.). *P. pannorum* metabolized nearly all carbon intermediates in the major fungal metabolic cycles ^13^ ( Figure 2). *P. destructans* utilized only a few simple sugars in glycolysis with no activity on a range of carbon intermediates. *P. pannorum* used a wider variety of nitrogen sources including amino acids, amino bases, and alkanes while *P. destructans* had a preference for the simple N sources and dipeptides ^13^ ( Figure 3). Most phosphorous sources tested supported the growth of *P. destructans* while *P. pannorum* only grew on few phosphosugars and phosphorylated nucleosides ( Figure 4). Both fungi did not utilize sulfur intermediates (Datasets 1–4 ^14^). Fifteen of ninety-five nutrient supplements supported good growth of *P. destructans* while *P. pannorum* grew only on D-Pantothenic acid ( Supplementary files). *P. pannorum* grew at very high salt concentrations and extreme acidic and basic pH ranges while *P. destructans* was sensitive to high salt (diminished growth ≥ 1% NaCl) and basic pH (diminished growth > pH 8.5) ( Figure 5). *P. pannorum* showed extreme tolerance to 96 xenobiotics in PM21, PM23 - PM25 plates in contrast to severe sensitivity observed in *P. destructans* (details not shown).

Excel sheets with OD _600_ values for all Biolog plates tested in this study Click here for additional data file. Copyright: © 2018 Chaturvedi V et al. 2018 Data associated with the article are available under the terms of the Creative Commons Zero "No rights reserved" data waiver (CC0 1.0 Public domain dedication).

## Discussion

Metabolic profiles of *P. destructans* and *P. pannorum* validated *in silico* predictions about the notable differences in the number of protein-encoding genes in their genomes ^11^. *P. destructans* contained enzymes and catabolic pathways that support fungal growth on a limited range of substrates of non-plant origin and showed high sensitivity to stress. *P. pannorum* was remarkably adapted for the nutrient poor environments of the caves and mines (‘extremophile’) with oligotrophic metabolism, osmotolerance, xerotolerance, and xenobiotic tolerance.

The findings in the present study confirm and expand on results from other reports on *P. destructans*’ adaptation and persistence in the North American caves and mines in the face of possible competitive interactions with the native fungal species ^8– 10^. Both Raudabaugh and Miller (2013) and Reynolds and Barton (2014) used a variety of biochemical tests to probe the metabolic activities in a collection of *Pseudogymnoascus* species isolates ^9, 10^. The authors of the former study surmised the suitability of *P. destructans* as a saprobe in the affected caves and mines in limited biotic competition (‘resource island’) ^10^. Reynolds and Barton (2014) found a reduced saprotrophic ability in *P. destructans* isolates vis-à-vis *P. pannorum* and other *Pseduogymnoascus* species, which suggested ‘co-evolution with the host’ ^9^. Wilson *et al.* (2017) performed a variety of tests including Biolog FF Microplate with 95 different substrates, and found limited saprotrophic ability in *P. destructans* in comparison to other *Pseudogymnoascus* species ^8^.

Further Phenotype Microarray profiling of *P. destructans* and *P. pannorum* would be crucial to fill-in current gaps in their genome sequences, define gene functions, and elucidate pathophysiological attributes ^11, 15, 16^.

The limitations of the current study include the use of single strains of two fungal species, and single end points instead of growth curves, which allow curve analysis for more accurate data interpretation as highlighted by other investigators.

We and others hope to accomplish these milestones with the recent availability of a high-quality *P. destructans* genome and data pipelines to automate Biolog analysis ^15, 17– 20^.

## Data availability

The data referenced by this article are under copyright with the following copyright statement: Copyright: © 2018 Chaturvedi V et al.

Data associated with the article are available under the terms of the Creative Commons Zero "No rights reserved" data waiver (CC0 1.0 Public domain dedication). 

Datasets 1–4: Excel sheets with OD _600_ values for all Biolog plates tested in this study. DOI, 10.5256/f1000research.15067.d204679 ^14^

## Supplementary material

**Supplementary File 1.** Biolog maps of PM 1 – 10, and PM 21, 23 – 25 plates
