# Supplementary material for: Phenotype profiling of white-nose syndrome pathogen Pseudogymnoascus destructans and closely-related Pseudogymnoascus pannorum reveals metabolic differences underlying fungal lifestyles
Source: F1000Res. 2018 Jul 17;7:665. Originally published 2018 May 25. [Version 2] doi: 10.12688/f1000research.15067.2 (PMC6039956; doi:10.12688/f1000research.15067.2)
Supplement: Supplementary file 2 [file f1000research-7-17010-s0001.tgz › d2173598-97a7-4973-8d93-e065dd841a49.zip!/PM1-PM10.pdf]

BIOLOG

Phenotype MicroArrays™

PM1 MicroPlate™ Carbon Sources

A1<br>Negative Control	A2<br>L-Arabinose	A3<br>N-Acetyl-D-Glucosamine	A4<br>D-Saccharic Acid	A5<br>Succinic Acid	A6<br>D-Galactose	A7<br>L-Aspartic Acid	A8<br>L-Proline	A9<br>D-Alanine	A10<br>D-Trehalose	A11<br>D-Mannose	A12<br>Dulcitol
B1<br>D-Serine	B2<br>D-Sorbitol	B3<br>Glycerol	B4<br>L-Fucose	B5<br>D-Glucuronic Acid	B6<br>D-Gluconic Acid	B7<br>D,L-α-Glycerol-Phosphate	B8<br>D-Xylose	B9<br>L-Lactic Acid	B10<br>Formic Acid	B11<br>D-Mannitol	B12<br>L-Glutamic Acid
C1<br>D-Glucose-6-Phosphate	C2<br>D-Galactonic Acid-γ-Lactone	C3<br>D,L-Malic Acid	C4<br>D-Ribose	C5<br>Tween 20	C6<br>L-Rhamnose	C7<br>D-Fructose	C8<br>Acetic Acid	C9<br>α-D-Glucose	C10<br>Maltose	C11<br>D-Melibiose	C12<br>Thymidine
D-1<br>L-Asparagine	D2<br>D-Aspartic Acid	D3<br>D-Glucosaminic Acid	D4<br>1,2-Propanediol	D5<br>Tween 40	D6<br>α-Keto-Glutaric Acid	D7<br>α-Keto-Butyric Acid	D8<br>α-Methyl-D-Galactoside	D9<br>α-D-Lactose	D10<br>Lactulose	D11<br>Sucrose	D12<br>Uridine
E1<br>L-Glutamine	E2<br>m-Tartaric Acid	E3<br>D-Glucose-1-Phosphate	E4<br>D-Fructose-6-Phosphate	E5<br>Tween 80	E6<br>α-Hydroxy Glutaric Acid-γ-Lactone	E7<br>α-Hydroxy Butyric Acid	E8<br>β-Methyl-D-Glucoside	E9<br>Adonitol	E10<br>Maltotriose	E11<br>2-Deoxy Adenosine	E12<br>Adenosine
F1<br>Glycyl-L-Aspartic Acid	F2<br>Citric Acid	F3<br>m-Inositol	F4<br>D-Threonine	F5<br>Fumaric Acid	F6<br>Bromo Succinic Acid	F7<br>Propionic Acid	F8<br>Mucic Acid	F9<br>Glycolic Acid	F10<br>Glyoxylic Acid	F11<br>D-Cellobiose	F12<br>Inosine
G1<br>Glycyl-L-Glutamic Acid	G2<br>Tricarballylic Acid	G3<br>L-Serine	G4<br>L-Threonine	G5<br>L-Alanine	G6<br>L-Alanyl-Glycine	G7<br>Acetoacetic Acid	G8<br>N-Acetyl-β-D-Mannosamine	G9<br>Mono Methyl Succinate	G10<br>Methyl Pyruvate	G11<br>D-Malic Acid	G12<br>L-Malic Acid
H1<br>Glycyl-L-Proline	H2<br>p-Hydroxy Phenyl Acetic Acid	H3<br>m-Hydroxy Phenyl Acetic Acid	H4<br>Tyramine	H5<br>D-Psicose	H6<br>L-Lyxose	H7<br>Glucuronamide	H8<br>Pyruvic Acid	H9<br>L-Galactonic Acid-γ-Lactone	H10<br>D-Galacturonic Acid	H11<br>Phenylethyl-amine	H12<br>2-Aminoethanol

PM2A MicroPlate™ Carbon Sources

A1<br>Negative Control	A2<br>Chondroitin Sulfate C	A3<br>α-Cyclodextrin	A4<br>β-Cyclodextrin	A5<br>γ-Cyclodextrin	A6<br>Dextrin	A7<br>Gelatin	A8<br>Glycogen	A9<br>Inulin	A10<br>Laminarin	A11<br>Mannan	A12<br>Pectin
B1<br>N-Acetyl-D-Galactosamine	B2<br>N-Acetyl-Neuraminic Acid	B3<br>β-D-Allose	B4<br>Amygdalin	B5<br>D-Arabinose	B6<br>D-Arabitol	B7<br>L-Arabitol	B8<br>Arbutin	B9<br>2-Deoxy-D-Ribose	B10<br>i-Erythritol	B11<br>D-Fucose	B12<br>3-0-β-D-Galacto-pyranosyl-D-Arabinose
C1<br>Gentiobiose	C2<br>L-Glucose	C3<br>Lactitol	C4<br>D-Melezitose	C5<br>Maltitol	C6<br>a-Methyl-D-Glucoside	C7<br>β-Methyl-D-Galactoside	C8<br>3-Methyl Glucose	C9<br>β-Methyl-D-Glucuronic Acid	C10<br>α-Methyl-D-Mannoside	C11<br>β-Methyl-D-Xyloside	C12<br>Palatinose
D1<br>D-Raffinose	D2<br>Salicin	D3<br>Sedoheptulosan	D4<br>L-Sorbose	D5<br>Stachyose	D6<br>D-Tagatose	D7<br>Turanose	D8<br>Xylitol	D9<br>N-Acetyl-D-Glucosaminitol	D10<br>γ-Amino Butyric Acid	D11<br>δ-Amino Valeric Acid	D12<br>Butyric Acid
E1<br>Capric Acid	E2<br>Caproic Acid	E3<br>Citraconic Acid	E4<br>Citramalic Acid	E5<br>D-Glucosamine	E6<br>2-Hydroxy Benzoic Acid	E7<br>4-Hydroxy Benzoic Acid	E8<br>β-Hydroxy Butyric Acid	E9<br>γ-Hydroxy Butyric Acid	E10<br>a-Keto-Valeric Acid	E11<br>Itaconic Acid	E12<br>5-Keto-D-Gluconic Acid
F1<br>D-Lactic Acid Methyl Ester	F2<br>Malonic Acid	F3<br>Melibionic Acid	F4<br>Oxalic Acid	F5<br>Oxalomalic Acid	F6<br>Quinic Acid	F7<br>D-Ribono-1,4-Lactone	F8<br>Sebacic Acid	F9<br>Sorbic Acid	F10<br>Succinamic Acid	F11<br>D-Tartaric Acid	F12<br>L-Tartaric Acid
G1<br>Acetamide	G2<br>L-Alaninamide	G3<br>N-Acetyl-L-Glutamic Acid	G4<br>L-Arginine	G5<br>Glycine	G6<br>L-Histidine	G7<br>L-Homoserine	G8<br>Hydroxy-L-Proline	G9<br>L-Isoleucine	G10<br>L-Leucine	G11<br>L-Lysine	G12<br>L-Methionine
H1<br>L-Ornithine	H2<br>L-Phenylalanine	H3<br>L-Pyroglutamic Acid	H4<br>L-Valine	H5<br>D,L-Carnitine	H6<br>Sec-Butylamine	H7<br>D.L-Octopamine	H8<br>Putrescine	H9<br>Dihydroxy Acetone	H10<br>2,3-Butanediol	H11<br>2,3-Butanedione	H12<br>3-Hydroxy 2-Butanone

PM3B MicroPlate™ Nitrogen Sources

**A1** Negative Control	**A2** Ammonia	**A3** Nitrite	**A4** Nitrate	**A5** Urea	**A6** Biuret	**A7** L-Alanine	**A8** L-Arginine	**A9** L-Asparagine	**A10** L-Aspartic Acid	**A11** L-Cysteine	**A12** L-Glutamic Acid
**B1** L-Glutamine	**B2** Glycine	**B3** L-Histidine	**B4** L-Isoleucine	**B5** L-Leucine	**B6** L-Lysine	**B7** L-Methionine	**B8** L-Phenylalanine	**B9** L-Proline	**B10** L-Serine	**B11** L-Threonine	**B12** L-Tryptophan
**C1** L-Tyrosine	**C2** L-Valine	**C3** D-Alanine	**C4** D-Asparagine	**C5** D-Aspartic Acid	**C6** D-Glutamic Acid	**C7** D-Lysine	**C8** D-Serine	**C9** D-Valine	**C10** L-Citrulline	**C11** L-Homoserine	**C12** L-Ornithine
**D-1** N-Acetyl-L-Glutamic Acid	**D2** N-Phthaloyl-L-Glutamic Acid	**D3** L-Pyroglutamic Acid	**D4** Hydroxylamine	**D5** Methylamine	**D6** N-Amylamine	**D7** N-Butylamine	**D8** Ethylamine	**D9** Ethanolamine	**D10** Ethylenediamine	**D11** Putrescine	**D12** Agmatine
**E1** Histamine	**E2** β-Phenylethyl-amine	**E3** Tyramine	**E4** Acetamide	**E5** Formamide	**E6** Glucuronamide	**E7** D,L-Lactamide	**E8** D-Glucosamine	**E9** D-Galactosamine	**E10** D-Mannosamine	**E11** N-Acetyl-D-Glucosamine	**E12** N-Acetyl-D-Galactosamine
**F1** N-Acetyl-D-Mannosamine	**F2** Adenine	**F3** Adenosine	**F4** Cytidine	**F5** Cytosine	**F6** Guanine	**F7** Guanosine	**F8** Thymine	**F9** Thymidine	**F10** Uracil	**F11** Uridine	**F12** Inosine
**G1** Xanthine	**G2** Xanthosine	**G3** Uric Acid	**G4** Alloxan	**G5** Allantoin	**G6** Parabanic Acid	**G7** D,L-α-Amino-N-Butyric Acid	**G8** γ-Amino-N-Butyric Acid	**G9** ε-Amino-N-Caproic Acid	**G10** D,L-α-Amino-Caprylic Acid	**G11** δ-Amino-N-Valeric Acid	**G12** α-Amino-N-Valeric Acid
**H1** Ala-Asp	**H2** Ala-Gln	**H3** Ala-Glu	**H4** Ala-Gly	**H5** Ala-His	**H6** Ala-Leu	**H7** Ala-Thr	**H8** Gly-Asn	**H9** Gly-Gln	**H10** Gly-Glu	**H11** Gly-Met	**H12** Met-Ala

PM4A MicroPlate™ Phosphorus and Sulfur Sources

**A1** Negative Control	**A2** Phosphate	**A3** Pyrophosphate	**A4** Trimeta-phosphate	**A5** Tripoly-phosphate	**A6** Triethyl Phosphate	**A7** Hypophosphite	**A8** Adenosine- 2'-monophosphate	**A9** Adenosine- 3'-monophosphate	**A10** Adenosine- 5'-monophosphate	**A11** Adenosine- 2',3'-cyclic monophosphate	**A12** Adenosine- 3',5'-cyclic monophosphate
**B1** Thiophosphate	**B2** Dithiophosphate	**B3** D,L-α-Glycerol Phosphate	**B4** β-Glycerol Phosphate	**B5** Carbamyl Phosphate	**B6** D-2-Phospho-Glyceric Acid	**B7** D-3-Phospho-Glyceric Acid	**B8** Guanosine- 2'-monophosphate	**B9** Guanosine- 3'-monophosphate	**B10** Guanosine- 5'-monophosphate	**B11** Guanosine- 2',3'-cyclic monophosphate	**B12** Guanosine- 3',5'-cyclic monophosphate
**C1** Phosphoenol Pyruvate	**C2** Phospho-Glycolic Acid	**C3** D-Glucose-1-Phosphate	**C4** D-Glucose-6-Phosphate	**C5** 2-Deoxy-D-Glucose 6-Phosphate	**C6** D-Glucosamine-6-Phosphate	**C7** 6-Phospho-Gluconic Acid	**C8** Cytidine- 2'-monophosphate	**C9** Cytidine- 3'-monophosphate	**C10** Cytidine- 5'-monophosphate	**C11** Cytidine- 2',3'-cyclic monophosphate	**C12** Cytidine- 3',5'-cyclic monophosphate
**D1** D-Mannose-1-Phosphate	**D2** D-Mannose-6-Phosphate	**D3** Cysteamine-S-Phosphate	**D4** Phospho-L-Arginine	**D5** O-Phospho-D-Serine	**D6** O-Phospho-L-Serine	**D7** O-Phospho-L-Threonine	**D8** Uridine- 2'-monophosphate	**D9** Uridine- 3'-monophosphate	**D10** Uridine- 5'-monophosphate	**D11** Uridine- 2',3'-cyclic monophosphate	**D12** Uridine- 3',5'-cyclic monophosphate
**E1** O-Phospho-D-Tyrosine	**E2** O-Phospho-L-Tyrosine	**E3** Phosphocreatine	**E4** Phosphoryl Choline	**E5** O-Phosphoryl-Ethanolamine	**E6** Phosphono Acetic Acid	**E7** 2-Aminoethyl Phosphonic Acid	**E8** Methylene Diphosphonic Acid	**E9** Thymidine- 3'-monophosphate	**E10** Thymidine- 5'-monophosphate	**E11** Inositol Hexaphosphate	**E12** Thymidine 3',5'-cyclic monophosphate
**F1** Negative Control	**F2** Sulfate	**F3** Thiosulfate	**F4** Tetrathionate	**F5** Thiophosphate	**F6** Dithiophosphate	**F7** L-Cysteine	**F8** D-Cysteine	**F9** L-Cysteinyl-Glycine	**F10** L-Cysteic Acid	**F11** Cysteamine	**F12** L-Cysteine Sulfinic Acid
**G1** N-Acetyl-L-Cysteine	**G2** S-Methyl-L-Cysteine	**G3** Cystathionine	**G4** Lanthionine	**G5** Glutathione	**G6** D,L-Ethionine	**G7** L-Methionine	**G8** D-Methionine	**G9** Glycyl-L-Methionine	**G10** N-Acetyl-D,L-Methionine	**G11** L- Methionine Sulfoxide	**G12** L-Methionine Sulfone
**H1** L-Djenkolic Acid	**H2** Thiourea	**H3** 1-Thio-β-D-Glucose	**H4** D,L-Lipoamide	**H5** Taurocholic Acid	**H6** Taurine	**H7** Hypotaurine	**H8** p-Amino Benzene Sulfonic Acid	**H9** Butane Sulfonic Acid	**H10** 2-Hydroxyethane Sulfonic Acid	**H11** Methane Sulfonic Acid	**H12** Tetramethylene Sulfone

PM5 MicroPlate™ Nutrient Supplements

A1<br>Negative Control	A2<br>Positive Control	A3<br>L-Alanine	A4<br>L-Arginine	A5<br>L-Asparagine	A6<br>L-Aspartic Acid	A7<br>L-Cysteine	A8<br>L-Glutamic Acid	A9<br>Adenosine-3',5'-cyclic monophosphate	A10<br>Adenine	A11<br>Adenosine	A12<br>2'-Deoxy Adenosine
B1<br>L-Glutamine	B2<br>Glycine	B3<br>L-Histidine	B4<br>L-Isoleucine	B5<br>L-Leucine	B6<br>L-Lysine	B7<br>L-Methionine	B8<br>L-Phenylalanine	B9<br>Guanosine-3',5'-cyclic monophosphate	B10<br>Guanine	B11<br>Guanosine	B12<br>2'-Deoxy Guanosine
C1<br>L-Proline	C2<br>L-Serine	C3<br>L-Threonine	C4<br>L-Tryptophan	C5<br>L-Tyrosine	C6<br>L-Valine	C7<br>L-isoleucine + L-Valine	C8<br>trans-4-Hydroxy L-Proline	C9<br>(5) 4-Amino-Imidazole-4(5)-Carboxamide	C10<br>Hypoxanthine	C11<br>Inosine	C12<br>2'-Deoxy Inosine
D1<br>L-Ornithine	D2<br>L-Citrulline	D3<br>Chorismic Acid	D4<br>(-)Shikimic Acid	D5<br>L-Homoserine Lactone	D6<br>D-Alanine	D7<br>D-Aspartic Acid	D8<br>D-Glutamic Acid	D9<br>D,L-α,ε-Diamino-pimelic Acid	D10<br>Cytosine	D11<br>Cytidine	D12<br>2'-Deoxy Cytidine
E1<br>Putrescine	E2<br>Spermidine	E3<br>Spermine	E4<br>Pyridoxine	E5<br>Pyridoxal	E6<br>Pyridoxamine	E7<br>β-Alanine	E8<br>D-Pantothenic Acid	E9<br>Orotic Acid	E10<br>Uracil	E11<br>Uridine	E12<br>2'-Deoxy Uridine
F1<br>Quinolinic Acid	F2<br>Nicotinic Acid	F3<br>Nicotinamide	F4<br>β-Nicotinamide Adenine Dinucleotide	F5<br>δ-Amino-Levulinic Acid	F6<br>Hematin	F7<br>Deferoxamine Mesylate	F8<br>D-(+)-Glucose	F9<br>N-Acetyl D-Glucosamine	F10<br>Thymine	F11<br>Glutathione (reduced form)	F12<br>Thymidine
G1<br>Oxaloacetic Acid	G2<br>D-Biotin	G3<br>Cyano-Cobalamine	G4<br>p-Amino-Benzoic Acid	G5<br>Folic Acid	G6<br>Inosine + Thiamine	G7<br>Thiamine	G8<br>Thiamine Pyrophosphate	G9<br>Riboflavin	G10<br>Pyrrolo-Quinoline Quinone	G11<br>Menadione	G12<br>m-Inositol
H1<br>Butyric Acid	H2<br>D,L-α-Hydroxy-Butyric Acid	H3<br>α-Keto-Butyric Acid	H4<br>Caprylic Acid	H5<br>D,L-α-Lipoic Acid (oxidized form)	H6<br>D,L-Mevalonic Acid	H7<br>D,L-Carnitine	H8<br>Choline	H9<br>Tween 20	H10<br>Tween 40	H11<br>Tween 60	H12<br>Tween 80

PM6 MicroPlate™ Peptide Nitrogen sources

A1<br>Negative Control	A2<br>Positive Control: L-Glutamine	A3<br>Ala-Ala	A4<br>Ala-Arg	A5<br>Ala-Asn	A6<br>Ala-Glu	A7<br>Ala-Gly	A8<br>Ala-His	A9<br>Ala-Leu	A10<br>Ala-Lys	A11<br>Ala-Phe	A12<br>Ala-Pro
B1<br>Ala-Ser	B2<br>Ala-Thr	B3<br>Ala-Trp	B4<br>Ala-Tyr	B5<br>Arg-Ala	B6<br>Arg-Arg	B7<br>Arg-Asp	B8<br>Arg-Gln	B9<br>Arg-Glu	B10<br>Arg-Ile	B11<br>Arg-Leu	B12<br>Arg-Lys
C1<br>Arg-Met	C2<br>Arg-Phe	C3<br>Arg-Ser	C4<br>Arg-Trp	C5<br>Arg-Tyr	C6<br>Arg-Val	C7<br>Asn-Glu	C8<br>Asn-Val	C9<br>Asp-Asp	C10<br>Asp-Glu	C11<br>Asp-Leu	C12<br>Asp-Lys
D1<br>Asp-Phe	D2<br>Asp-Trp	D3<br>Asp-Val	D4<br>Cys-Gly	D5<br>Gln-Gln	D6<br>Gln-Gly	D7<br>Glu-Asp	D8<br>Glu-Glu	D9<br>Glu-Gly	D10<br>Glu-Ser	D11<br>Glu-Trp	D12<br>Glu-Tyr
E1<br>Glu-Val	E2<br>Gly-Ala	E3<br>Gly-Arg	E4<br>Gly-Cys	E5<br>Gly-Gly	E6<br>Gly-His	E7<br>Gly-Leu	E8<br>Gly-Lys	E9<br>Gly-Met	E10<br>Gly-Phe	E11<br>Gly-Pro	E12<br>Gly-Ser
F1<br>Gly-Thr	F2<br>Gly-Trp	F3<br>Gly-Tyr	F4<br>Gly-Val	F5<br>His-Asp	F6<br>His-Gly	F7<br>His-Leu	F8<br>His-Lys	F9<br>His-Met	F10<br>His-Pro	F11<br>His-Ser	F12<br>His-Trp
G1<br>His-Tyr	G2<br>His-Val	G3<br>Ile-Ala	G4<br>Ile-Arg	G5<br>Ile-Gln	G6<br>Ile-Gly	G7<br>Ile-His	G8<br>Ile-Ile	G9<br>Ile-Met	G10<br>Ile-Phe	G11<br>Ile-Pro	G12<br>Ile-Ser
H1<br>Ile-Trp	H2<br>Ile-Tyr	H3<br>Ile-Val	H4<br>Leu-Ala	H5<br>Leu-Arg	H6<br>Leu-Asp	H7<br>Leu-Glu	H8<br>Leu-Gly	H9<br>Leu-Ile	H10<br>Leu-Leu	H11<br>Leu-Met	H12<br>Leu-Phe

PM7 MicroPlate™ Peptide Nitrogen sources

A1<br>Negative Control	A2<br>Positive Control: L-Glutamine	A3<br>Leu-Ser	A4<br>Leu-Trp	A5<br>Leu-Val	A6<br>Lys-Ala	A7<br>Lys-Arg	A8<br>Lys-Glu	A9<br>Lys-Ile	A10<br>Lys-Leu	A11<br>Lys-Lys	A12<br>Lys-Phe
B1<br>Lys-Pro	B2<br>Lys-Ser	B3<br>Lys-Thr	B4<br>Lys-Trp	B5<br>Lys-Tyr	B6<br>Lys-Val	B7<br>Met-Arg	B8<br>Met-Asp	B9<br>Met-Gln	B10<br>Met-Glu	B11<br>Met-Gly	B12<br>Met-His
C1<br>Met-Ile	C2<br>Met-Leu	C3<br>Met-Lys	C4<br>Met-Met	C5<br>Met-Phe	C6<br>Met-Pro	C7<br>Met-Trp	C8<br>Met-Val	C9<br>Phe-Ala	C10<br>Phe-Gly	C11<br>Phe-Ile	C12<br>Phe-Phe
D1<br>Phe-Pro	D2<br>Phe-Ser	D3<br>Phe-Trp	D4<br>Pro-Ala	D5<br>Pro-Asp	D6<br>Pro-Gln	D7<br>Pro-Gly	D8<br>Pro-Hyp	D9<br>Pro-Leu	D10<br>Pro-Phe	D11<br>Pro-Pro	D12<br>Pro-Tyr
E1<br>Ser-Ala	E2<br>Ser-Gly	E3<br>Ser-His	E4<br>Ser-Leu	E5<br>Ser-Met	E6<br>Ser-Phe	E7<br>Ser-Pro	E8<br>Ser-Ser	E9<br>Ser-Tyr	E10<br>Ser-Val	E11<br>Thr-Ala	E12<br>Thr-Arg
F1<br>Thr-Glu	F2<br>Thr-Gly	F3<br>Thr-Leu	F4<br>Thr-Met	F5<br>Thr-Pro	F6<br>Trp-Ala	F7<br>Trp-Arg	F8<br>Trp-Asp	F9<br>Trp-Glu	F10<br>Trp-Gly	F11<br>Trp-Leu	F12<br>Trp-Lys
G1<br>Trp-Phe	G2<br>Trp-Ser	G3<br>Trp-Trp	G4<br>Trp-Tyr	G5<br>Tyr-Ala	G6<br>Tyr-Gln	G7<br>Tyr-Glu	G8<br>Tyr-Gly	G9<br>Tyr-His	G10<br>Tyr-Leu	G11<br>Tyr-Lys	G12<br>Tyr-Phe
H1<br>Tyr-Trp	H2<br>Tyr-Tyr	H3<br>Val-Arg	H4<br>Val-Asn	H5<br>Val-Asp	H6<br>Val-Gly	H7<br>Val-His	H8<br>Val-Ile	H9<br>Val-Leu	H10<br>Val-Tyr	H11<br>Val-Val	H12<br>ϒ-Glu-Gly

PM8 MicroPlate™ Peptide Nitrogen sources

A1<br>Negative Control	A2<br>Positive Control: L-Glutamine	A3<br>Ala-Asp	A4<br>Ala-Gln	A5<br>Ala-Ile	A6<br>Ala-Met	A7<br>Ala-Val	A8<br>Asp-Ala	A9<br>Asp-Gln	A10<br>Asp-Gly	A11<br>Glu-Ala	A12<br>Gly-Asn
B1<br>Gly-Asp	B2<br>Gly-Ile	B3<br>His-Ala	B4<br>His-Glu	B5<br>His-His	B6<br>Ile-Asn	B7<br>Ile-Leu	B8<br>Leu-Asn	B9<br>Leu-His	B10<br>Leu-Pro	B11<br>Leu-Tyr	B12<br>Lys-Asp
C1<br>Lys-Gly	C2<br>Lys-Met	C3<br>Met-Thr	C4<br>Met-Tyr	C5<br>Phe-Asp	C6<br>Phe-Glu	C7<br>Gln-Glu	C8<br>Phe-Met	C9<br>Phe-Tyr	C10<br>Phe-Val	C11<br>Pro-Arg	C12<br>Pro-Asn
D1<br>Pro-Glu	D2<br>Pro-Ile	D3<br>Pro-Lys	D4<br>Pro-Ser	D5<br>Pro-Trp	D6<br>Pro-Val	D7<br>Ser-Asn	D8<br>Ser-Asp	D9<br>Ser-Gln	D10<br>Ser-Glu	D11<br>Thr-Asp	D12<br>Thr-Gln
E1<br>Thr-Phe	E2<br>Thr-Ser	E3<br>Trp-Val	E4<br>Tyr-Ile	E5<br>Tyr-Val	E6<br>Val-Ala	E7<br>Val-Gln	E8<br>Val-Glu	E9<br>Val-Lys	E10<br>Val-Met	E11<br>Val-Phe	E12<br>Val-Pro
F1<br>Val-Ser	F2<br>β-Ala-Ala	F3<br>β-Ala-Gly	F4<br>β-Ala-His	F5<br>Met-β-Ala	F6<br>β-Ala-Phe	F7<br>D-Ala-D-Ala	F8<br>D-Ala-Gly	F9<br>D-Ala-Leu	F10<br>D-Leu-D-Leu	F11<br>D-Leu-Gly	F12<br>D-Leu-Tyr
G1<br>ϒ-Glu-Gly	G2<br>ϒ-D-Glu-Gly	G3<br>Gly-D-Ala	G4<br>Gly-D-Asp	G5<br>Gly-D-Ser	G6<br>Gly-D-Thr	G7<br>Gly-D-Val	G8<br>Leu-β-Ala	G9<br>Leu-D-Leu	G10<br>Phe-β-Ala	G11<br>Ala-Ala-Ala	G12<br>D-Ala-Gly-Gly
H1<br>Gly-Gly-Ala	H2<br>Gly-Gly-D-Leu	H3<br>Gly-Gly-Gly	H4<br>Gly-Gly-Ile	H5<br>Gly-Gly-Leu	H6<br>Gly-Gly-Phe	H7<br>Val-Tyr-Val	H8<br>Gly-Phe-Phe	H9<br>Leu-Gly-Gly	H10<br>Leu-Leu-Leu	H11<br>Phe-Gly-Gly	H12<br>Tyr-Gly-Gly

PM9 MicroPlate™ Osmolytes

A1<br>NaCl 1%	A2<br>NaCl 2%	A3<br>NaCl 3%	A4<br>NaCl 4%	A5<br>NaCl 5%	A6<br>NaCl 5.5%	A7<br>NaCl 6%	A8<br>NaCl 6.5%	A9<br>NaCl 7%	A10<br>NaCl 8%	A11<br>NaCl 9%	A12<br>NaCl 10%
B1<br>NaCl 6%	B2<br>NaCl 6% +<br>Betaine	B3<br>NaCl 6% +<br>N-N Dimethyl<br>Glycine	B4<br>NaCl 6% +<br>Sarcosine	B5<br>NaCl 6% +<br>Dimethyl<br>sulphonyl<br>propionate	B6<br>NaCl 6% +<br>MOPS	B7<br>NaCl 6% +<br>Ectoine	B8<br>NaCl 6% +<br>Choline	B9<br>NaCl 6% +<br>Phosphoryl<br>Choline	B10<br>NaCl 6% +<br>Creatine	B11<br>NaCl 6% +<br>Creatinine	B12<br>NaCl 6% +<br>L- Carnitine
C1<br>NaCl 6% +<br>KCl	C2<br>NaCl 6% +<br>L-Proline	C3<br>NaCl 6% +<br>N-Acetyl<br>L-Glutamine	C4<br>NaC1 6% +<br>β-Glutamic Acid	C5<br>NaC1 6% +<br>γ–Amino -N-<br>Butyric Acid	C6<br>NaC1 6% +<br>Glutathione	C7<br>NaCl 6% +<br>Glycerol	C8<br>NaC1 6% +<br>Trehalose	C9<br>NaC1 6% +<br>Trimethylamine-<br>N-oxide	C10<br>NaC1 6% +<br>Trimethylamine	C11<br>NaCl 6% +<br>Octopine	C12<br>NaC1 6% +<br>Trigonelline
D1<br>Potassium<br>chloride<br>3%	D2<br>Potassium<br>chloride<br>4%	D3<br>Potassium<br>chloride<br>5%	D4<br>Potassium<br>chloride<br>6%	D5<br>Sodium sulfate<br>2%	D6<br>Sodium sulfate<br>3%	D7<br>Sodium sulfate<br>4%	D8<br>Sodium sulfate<br>5%	D9<br>Ethylene glycol<br>5%	D10<br>Ethylene glycol<br>10%	D11<br>Ethylene glycol<br>15%	D12<br>Ethylene glycol<br>20%
E1<br>Sodium formate<br>1%	E2<br>Sodium formate<br>2%	E3<br>Sodium formate<br>3%	E4<br>Sodium formate<br>4%	E5<br>Sodium formate<br>5%	E6<br>Sodium formate<br>6%	E7<br>Urea<br>2%	E8<br>Urea<br>3%	E9<br>Urea<br>4%	E10<br>Urea<br>5%	E11<br>Urea<br>6%	E12<br>Urea<br>7%
F1<br>Sodium Lactate<br>1%	F2<br>Sodium Lactate<br>2%	F3<br>Sodium Lactate<br>3%	F4<br>Sodium Lactate<br>4%	F5<br>Sodium Lactate<br>5%	F6<br>Sodium Lactate<br>6%	F7<br>Sodium Lactate<br>7%	F8<br>Sodium Lactate<br>8%	F9<br>Sodium Lactate<br>9%	F10<br>Sodium Lactate<br>10%	F11<br>Sodium Lactate<br>11%	F12<br>Sodium Lactate<br>12%
G1<br>Sodium<br>Phosphate pH 7<br>20mM	G2<br>Sodium<br>Phosphate pH 7<br>50mM	G3<br>Sodium<br>Phosphate pH 7<br>100mM	G4<br>Sodium<br>Phosphate pH 7<br>200mM	G5<br>Sodium<br>Benzoate pH 5.2<br>20mM	G6<br>Sodium<br>Benzoate pH 5.2<br>50mM	G7<br>Sodium<br>Benzoate pH5.2<br>100mM	G8<br>Sodium<br>Benzoate pH 5.2<br>200mM	G9<br>Ammonium<br>sulfate pH8<br>10mM	G10<br>Ammonium<br>sulfate pH 8<br>20mM	G11<br>Ammonium<br>sulfate pH 8<br>50mM	G12<br>Ammonium<br>sulfate pH8<br>100mM
H1<br>Sodium Nitrate<br>10mM	H2<br>Sodium Nitrate<br>20mM	H3<br>Sodium Nitrate<br>40mM	H4<br>Sodium Nitrate<br>60mM	H5<br>Sodium Nitrate<br>80mM	H6<br>Sodium Nitrate<br>100mM	H7<br>Sodium Nitrite<br>10mM	H8<br>Sodium Nitrite<br>20mM	H9<br>Sodium Nitrite<br>40mM	H10<br>Sodium Nitrite<br>60mM	H11<br>Sodium Nitrite<br>80mM	H12<br>Sodium Nitrite<br>100mM

PM10 MicroPlate™ pH

A1<br>pH 3.5	A2<br>pH 4	A3<br>pH 4.5	A4<br>pH 5	A5<br>pH 5.5	A6<br>pH 6	A7<br>pH 7	A8<br>pH 8	A9<br>pH 8.5	A10<br>pH 9	A11<br>pH 9.5	A12<br>pH 10
B1<br>pH 4.5	B2<br>pH 4.5 +<br>L-Alanine	B3<br>pH 4.5 +<br>L-Arginine	B4<br>pH 4.5 +<br>L-Asparagine	B5<br>pH 4.5 +<br>L-Aspartic Acid	B6<br>pH 4.5 +<br>L-Glutamic Acid	B7<br>pH 4.5 +<br>L-Glutamine	B8<br>pH 4.5 +<br>Glycine	B9<br>pH 4.5 +<br>L-Histidine	B10<br>pH 4.5 +<br>L-Isoleucine	B11<br>pH 4.5 +<br>L-Leucine	B12<br>pH 4.5 +<br>L-Lysine
C1<br>pH 4.5 +<br>L-Methionine	C2<br>pH 4.5 +<br>L-Phenylalanine	C3<br>pH 4.5 +<br>L-Proline	C4<br>pH 4.5 +<br>L-Serine	C5<br>pH 4.5 +<br>L-Threonine	C6<br>pH 4.5 +<br>L-Tryptophan	C7<br>pH 4.5 +<br>L-Citrulline	C8<br>pH 4.5 +<br>L-Valine	C9<br>pH 4.5 +<br>Hydroxy-<br>L-Proline	C10<br>pH 4.5 +<br>L-Ornithine	C11<br>pH 4.5 +<br>L-Homoarginine	C12<br>pH 4.5 +<br>L-Homoserine
D-1<br>pH 4.5 +<br>Anthranilic Acid	D2<br>pH 4.5 +<br>L-Norleucine	D3<br>pH 4.5 +<br>L-Norvaline	D4<br>pH 4.5 +<br>α- Amino-N-<br>Butyric Acid	D5<br>pH 4.5 +<br>p-Amino-<br>Benzoic Acid	D6<br>pH 4.5 +<br>L-Cysteic Acid	D7<br>pH 4.5 +<br>D-Lysine	D8<br>pH 4.5 +<br>5-Hydroxy<br>Lysine	D9<br>pH 4.5 +<br>5-Hydroxy<br>Tryptophan	D10<br>pH 4.5 +<br>D,L-Diamino<br>pimelic Acid	D11<br>pH 4.5 +<br>Trimethylamine-<br>N-oxide	D12<br>pH 4.5 +<br>Urea
E1<br>pH 9.5	E2<br>pH 9.5 +<br>L-Alanine	E3<br>pH 9.5 +<br>L-Arginine	E4<br>pH 9.5 +<br>L-Asparagine	E5<br>pH 9.5 +<br>L-Aspartic Acid	E6<br>pH 9.5 +<br>L-Glutamic Acid	E7<br>pH 9.5 +<br>L-Glutamine	E8<br>pH 9.5 +<br>Glycine	E9<br>pH 9.5 +<br>L-Histidine	E10<br>pH 9.5 +<br>L-Isoleucine	E11<br>pH 9.5 +<br>L-Leucine	E12<br>pH 9.5 +<br>L-Lysine
F1<br>pH 9.5 +<br>L-Methionine	F2<br>pH 9.5 +<br>L-Phenylalanine	F3<br>pH 9.5 +<br>L-Proline	F4<br>pH 9.5 +<br>L-Serine	F5<br>pH 9.5 +<br>L-Threonine	F6<br>pH 9.5 +<br>L-Tryptophan	F7<br>pH 9.5 +<br>L-Tyrosine	F8<br>pH 9.5 +<br>L-Valine	F9<br>pH 9.5 +<br>Hydroxy-<br>L-Proline	F10<br>pH 9.5 +<br>L-Ornithine	F11<br>pH 9.5 +<br>L-Homoarginine	F12<br>pH 9.5 +<br>L-Homoserine
G1<br>pH 9.5 +<br>Anthranilic acid	G2<br>pH 9.5 +<br>L-Norleucine	G3<br>pH 9.5 +<br>L-Norvaline	G4<br>pH 9.5 +<br>Agmatine	G5<br>pH 9.5 +<br>Cadaverine	G6<br>pH 9.5 +<br>Putrescine	G7<br>pH 9.5 +<br>Histamine	G8<br>pH 9.5 +<br>Phenylethylamine	G9<br>pH 9.5 +<br>Tyramine	G10<br>pH 9.5 +<br>Creatine	G11<br>pH 9.5 +<br>Trimethylamine-<br>N-oxide	G12<br>pH 9.5 +<br>Urea
H1<br>X-Caprylate	H2<br>X–α-D-<br>Glucoside	H3<br>X-β-D-<br>Glucoside	H4<br>X-α-D-<br>Galactoside	H5<br>X-β-D-<br>Galactoside	H6<br>X-α- D-<br>Glucuronide	H7<br>X-β- D-<br>Glucuronide	H8<br>X-β-D-<br>Glucosaminide	H9<br>X-β-D-<br>Galactosaminide	H10<br>X-α-D-<br>Mannoside	H11<br>X-PO4	H12<br>X-SO4
