# Supplementary material for: Phenotype profiling of white-nose syndrome pathogen Pseudogymnoascus destructans and closely-related Pseudogymnoascus pannorum reveals metabolic differences underlying fungal lifestyles
Source: F1000Res. 2018 Jul 17;7:665. Originally published 2018 May 25. [Version 2] doi: 10.12688/f1000research.15067.2 (PMC6039956; doi:10.12688/f1000research.15067.2)
Supplement: Supplementary file 2 [file f1000research-7-17010-s0001.tgz › d2173598-97a7-4973-8d93-e065dd841a49.zip!/PM21-PM25.pdf]

BIOLOG

Phenotype MicroArrays™

PM21D MicroPlate™ Chemical Sensitivity

A1 Guanidine hydrochloride	A2 Guanidine hydrochloride	A3 Guanidine hydrochloride	A4 Guanidine hydrochloride	A5 2,2`-Dipyridyl	A6 2,2`-Dipyridyl	A7 2,2`-Dipyridyl	A8 2,2`-Dipyridyl	A9 Promethazine	A10 Promethazine	A11 Promethazine	A12 Promethazine
B1 Nystatin	B2 Nystatin	B3 Nystatin	B4 Nystatin	B5 Dodecyltrimethyl ammonium bromide	B6 Dodecyltrimethyl ammonium bromide	B7 Dodecyltrimethyl ammonium bromide	B8 Dodecyltrimethyl ammonium bromide	B9 Protamine sulfate	B10 Protamine sulfate	B11 Protamine sulfate	B12 Protamine sulfate
C1 Cetylpyridinium chloride	C2 Cetylpyridinium chloride	C3 Cetylpyridinium chloride	C4 Cetylpyridinium chloride	C5 Domiphen bromide	C6 Domiphen bromide	C7 Domiphen bromide	C8 Domiphen bromide	C9 L-Aspartic acid b-hydroxamate	C10 L-Aspartic acid b-hydroxamate	C11 L-Aspartic acid b-hydroxamate	C12 L-Aspartic acid b-hydroxamate
D1 1-Hydroxypyridine-2-thione	D2 1-Hydroxypyridine-2-thione	D3 1-Hydroxypyridine-2-thione	D4 1-Hydroxypyridine-2-thione	D5 EDTA	D6 EDTA	D7 EDTA	D8 EDTA	D9 Sodium dichromate	D10 Sodium dichromate	D11 Sodium dichromate	D12 Sodium dichromate
E1 Compound 48/80	E2 Compound 48/80	E3 Compound 48/80	E4 Compound 48/80	E5 Manganese(II) chloride	E6 Manganese(II) chloride	E7 Manganese(II) chloride	E8 Manganese(II) chloride	E9 Magnesium chloride	E10 Magnesium chloride	E11 Magnesium chloride	E12 Magnesium chloride
F1 Copper(II) sulfate	F2 Copper(II) sulfate	F3 Copper(II) sulfate	F4 Copper(II) sulfate	F5 Neomycin	F6 Neomycin	F7 Neomycin	F8 Neomycin	F9 D-Cycloserine	F10 D-Cycloserine	F11 D-Cycloserine	F12 D-Cycloserine
G1 Sodium Selenite	G2 Sodium Selenite	G3 Sodium Selenite	G4 Sodium Selenite	G5 Nickel chloride	G6 Nickel chloride	G7 Nickel chloride	G8 Nickel chloride	G9 Trifluoperazine	G10 Trifluoperazine	G11 Trifluoperazine	G12 Trifluoperazine
H1 Diamide	H2 Diamide	H3 Diamide	H4 Diamide	H5 Thiourea	H6 Thiourea	H7 Thiourea	H8 Thiourea	H9 Zinc chloride	H10 Zinc chloride	H11 Zinc chloride	H12 Zinc chloride

PM22D MicroPlate™ Chemical Sensitivity

A1 L-Glutamic acid g-hydroxamate	A2 L-Glutamic acid g-hydroxamate	A3 L-Glutamic acid g-hydroxamate	A4 L-Glutamic acid g-hydroxamate	A5 Sodium metavanadate	A6 Sodium metavanadate	A7 Sodium metavanadate	A8 Sodium metavanadate	A9 Caffeine	A10 Caffeine	A11 Caffeine	A12 Caffeine
B1 L-Arginine hydroxamate	B2 L-Arginine hydroxamate	B3 L-Arginine hydroxamate	B4 L-Arginine hydroxamate	B5 Glycine hydroxamate	B6 Glycine hydroxamate	B7 Glycine hydroxamate	B8 Glycine hydroxamate	B9 Triclosan	B10 Triclosan	B11 Triclosan	B12 Triclosan
C1 3-Amino-1,2,4-triazole	C2 3-Amino-1,2,4-triazole	C3 3-Amino-1,2,4-triazole	C4 3-Amino-1,2,4-triazole	C5 Miltefosine	C6 Miltefosine	C7 Miltefosine	C8 Miltefosine	C9 D,L-Serine hydroxamate	C10 D,L-Serine hydroxamate	C11 D,L-Serine hydroxamate	C12 D,L-Serine hydroxamate
D1 Polymyxin B	D2 Polymyxin B	D3 Polymyxin B	D4 Polymyxin B	D5 Urea hydrogen peroxide	D6 Urea hydrogen peroxide	D7 Urea hydrogen peroxide	D8 Urea hydrogen peroxide	D9 CCCP	D10 CCCP	D11 CCCP	D12 CCCP
E1 FCCP	E2 FCCP	E3 FCCP	E4 FCCP	E5 BAPTA	E6 BAPTA	E7 BAPTA	E8 BAPTA	E9 D-Serine	E10 D-Serine	E11 D-Serine	E12 D-Serine
F1 Azaserine	F2 Azaserine	F3 Azaserine	F4 Azaserine	F5 Lithium chloride	F6 Lithium chloride	F7 Lithium chloride	F8 Lithium chloride	F9 Bleomycin	F10 Bleomycin	F11 Bleomycin	F12 Bleomycin
G1 Benzamidine	G2 Benzamidine	G3 Benzamidine	G4 Benzamidine	G5 Cycloheximide	G6 Cycloheximide	G7 Cycloheximide	G8 Cycloheximide	G9 Thallium(I) acetate	G10 Thallium(I) acetate	G11 Thallium(I) acetate	G12 Thallium(I) acetate
H1 Bleomycin	H2 Bleomycin	H3 Bleomycin	H4 Bleomycin	H5 Paromomycin	H6 Paromomycin	H7 Paromomycin	H8 Paromomycin	H9 Myclobutanil	H10 Myclobutanil	H11 Myclobutanil	H12 Myclobutanil

Phenotype MicroArrays™

PM23A MicroPlate™ Chemical Sensitivity

A1 Benzethonium Chloride	A2 Benzethonium Chloride	A3 Benzethonium Chloride	A4 Benzethonium Chloride	A5 Chlorpromazine hydrochloride	A6 Chlorpromazine hydrochloride	A7 Chlorpromazine hydrochloride	A8 Chlorpromazine hydrochloride	A9 Ammonium Sulfate	A10 Ammonium Sulfate	A11 Ammonium Sulfate	A12 Ammonium Sulfate
B1 Cadmium Chloride hydrate	B2 Cadmium Chloride hydrate	B3 Cadmium Chloride hydrate	B4 Cadmium Chloride hydrate	B5 Dequalinium Chloride	B6 Dequalinium Chloride	B7 Dequalinium Chloride	B8 Dequalinium Chloride	B9 Doxycycline Hyclate	B10 Doxycycline Hyclate	B11 Doxycycline Hyclate	B12 Doxycycline Hyclate
C1 Glycine hydrochloride	C2 Glycine hydrochloride	C3 Glycine hydrochloride	C4 Glycine hydrochloride	C5 Hydroxylamine hydrochloride	C6 Hydroxylamine hydrochloride	C7 Hydroxylamine hydrochloride	C8 Hydroxylamine hydrochloride	C9 Poly-L-lysine hydrochloride	C10 Poly-L-lysine hydrochloride	C11 Poly-L-lysine hydrochloride	C12 Poly-L-lysine hydrochloride
D1 Chromium(III) Chloride hexahydrate	D2 Chromium(III) Chloride hexahydrate	D3 Chromium(III) Chloride hexahydrate	D4 Chromium(III) Chloride hexahydrate	D5 Cobalt(II) Chloride hexahydrate	D6 Cobalt(II) Chloride hexahydrate	D7 Cobalt(II) Chloride hexahydrate	D8 Cobalt(II) Chloride hexahydrate	D9 Cupric Chloride Dihydrate	D10 Cupric Chloride Dihydrate	D11 Cupric Chloride Dihydrate	D12 Cupric Chloride Dihydrate
E1 Sodium metaborate Tetrahydrate	E2 Sodium metaborate Tetrahydrate	E3 Sodium metaborate Tetrahydrate	E4 Sodium metaborate Tetrahydrate	E5 Sodium (meta)periodate	E6 Sodium (meta)periodate	E7 Sodium (meta)periodate	E8 Sodium (meta)periodate	E9 Sodium Arsenite	E10 Sodium Arsenite	E11 Sodium Arsenite	E12 Sodium Arsenite
F1 Sodium Azide	F2 Sodium Azide	F3 Sodium Azide	F4 Sodium Azide	F5 Sodium Caprylate	F6 Sodium Caprylate	F7 Sodium Caprylate	F8 Sodium Caprylate	F9 Sodium Cyanate	F10 Sodium Cyanate	F11 Sodium Cyanate	F12 Sodium Cyanate
G1 Sodium Nitrite	G2 Sodium Nitrite	G3 Sodium Nitrite	G4 Sodium Nitrite	G5 Sodium Orthovanadate	G6 Sodium Orthovanadate	G7 Sodium Orthovanadate	G8 Sodium Orthovanadate	G9 2-Deoxy-D-glucose	G10 2-Deoxy-D-glucose	G11 2-Deoxy-D-glucose	G12 2-Deoxy-D-glucose
H1 Sodium Selenate	H2 Sodium Selenate	H3 Sodium Selenate	H4 Sodium Selenate	H5 Sodium Cyanide	H6 Sodium Cyanide	H7 Sodium Cyanide	H8 Sodium Cyanide	H9 Sodium Thiosulfate	H10 Sodium Thiosulfate	H11 Sodium Thiosulfate	H12 Sodium Thiosulfate

PM24C MicroPlate™ Chemical Sensitivity

A1 Apramycin Sulfate	A2 Apramycin Sulfate	A3 Apramycin Sulfate	A4 Apramycin Sulfate	A5 Aminacrine	A6 Aminacrine	A7 Aminacrine	A8 Aminacrine	A9 Zaragozic acid A	A10 Zaragozic acid A	A11 Zaragozic acid A	A12 Zaragozic acid A
B1 Blasticidin hydrochloride	B2 Blasticidin hydrochloride	B3 Blasticidin hydrochloride	B4 Blasticidin hydrochloride	B5 Thioridazine hydrochloride	B6 Thioridazine hydrochloride	B7 Thioridazine hydrochloride	B8 Thioridazine hydrochloride	B9 Sodium Benzoate	B10 Sodium Benzoate	B11 Sodium Benzoate	B12 Sodium Benzoate
C1 Chlortetracycline hydrochloride	C2 Chlortetracycline hydrochloride	C3 Chlortetracycline hydrochloride	C4 Chlortetracycline hydrochloride	C5 Sodium metasilicate	C6 Sodium metasilicate	C7 Sodium metasilicate	C8 Sodium metasilicate	C9 Pentamidine Isethionate	C10 Pentamidine Isethionate	C11 Pentamidine Isethionate	C12 Pentamidine Isethionate
D1 6-Azauracil	D2 6-Azauracil	D3 6-Azauracil	D4 6-Azauracil	D5 Potassium Chromate	D6 Potassium Chromate	D7 Potassium Chromate	D8 Potassium Chromate	D9 Thialysine	D10 Thialysine	D11 Thialysine	D12 Thialysine
E1 Berberine	E2 Berberine	E3 Berberine	E4 Berberine	E5 EGTA	E6 EGTA	E7 EGTA	E8 EGTA	E9 Sodium Pyrophosphate Decahydrate	E10 Sodium Pyrophosphate Decahydrate	E11 Sodium Pyrophosphate Decahydrate	E12 Sodium Pyrophosphate Decahydrate
F1 Isoniazid	F2 Isoniazid	F3 Isoniazid	F4 Isoniazid	F5 Methyl Viologen Dichloride hydrate	F6 Methyl Viologen Dichloride hydrate	F7 Methyl Viologen Dichloride hydrate	F8 Methyl Viologen Dichloride hydrate	F9 Sodium Fluoride	F10 Sodium Fluoride	F11 Sodium Fluoride	F12 Sodium Fluoride
G1 Cisplatin	G2 Cisplatin	G3 Cisplatin	G4 Cisplatin	G5 Aluminum sulfate	G6 Aluminum sulfate	G7 Aluminum sulfate	G8 Aluminum sulfate	G9 Fluconazole	G10 Fluconazole	G11 Fluconazole	G12 Fluconazole
H1 Propiconazole	H2 Propiconazole	H3 Propiconazole	H4 Propiconazole	H5 Tamoxifen	H6 Tamoxifen	H7 Tamoxifen	H8 Tamoxifen	H9 Miconazole Nitrate	H10 Miconazole Nitrate	H11 Miconazole Nitrate	H12 Miconazole Nitrate

BIOLOG

Phenotype MicroArrays™

PM25D MicroPlate™ Chemical Sensitivity

A1<br>Hydroxyurea	A2<br>Hydroxyurea	A3<br>Hydroxyurea	A4<br>Hydroxyurea	A5<br>Tobramycin	A6<br>Tobramycin	A7<br>Tobramycin	A8<br>Tobramycin	A9<br>Niaproof	A10<br>Niaproof	A11<br>Niaproof	A12<br>Niaproof
B1<br>Chloroalanine hydrochloride	B2<br>Chloroalanine hydrochloride	B3<br>Chloroalanine hydrochloride	B4<br>Chloroalanine hydrochloride	B5<br>Tetrazolium Violet	B6<br>Tetrazolium Violet	B7<br>Tetrazolium Violet	B8<br>Tetrazolium Violet	B9<br>Kanamycin Monosulfate	B10<br>Kanamycin Monosulfate	B11<br>Kanamycin Monosulfate	B12<br>Kanamycin Monosulfate
C1<br>4-Aminopyridine	C2<br>4-Aminopyridine	C3<br>4-Aminopyridine	C4<br>4-Aminopyridine	C5<br>Amitriptyline hydrochloride	C6<br>Amitriptyline hydrochloride	C7<br>Amitriptyline hydrochloride	C8<br>Amitriptyline hydrochloride	C9<br>4-Nitroquinoline-N-Oxide	C10<br>4-Nitroquinoline-N-Oxide	C11<br>4-Nitroquinoline-N-Oxide	C12<br>4-Nitroquinoline-N-Oxide
D1<br>Alexidine	D2<br>Alexidine	D3<br>Alexidine	D4<br>Alexidine	D5<br>Hygromycin B	D6<br>Hygromycin B	D7<br>Hygromycin B	D8<br>Hygromycin B	D9<br>Fluorodeoxyuridine	D10<br>Fluorodeoxyuridine	D11<br>Fluorodeoxyuridine	D12<br>Fluorodeoxyuridine
E1<br>Sodium salicylate	E2<br>Sodium salicylate	E3<br>Sodium salicylate	E4<br>Sodium salicylate	E5<br>Succinic acid	E6<br>Succinic acid	E7<br>Succinic acid	E8<br>Succinic acid	E9<br>Clomiphene Citrate	E10<br>Clomiphene Citrate	E11<br>Clomiphene Citrate	E12<br>Clomiphene Citrate
F1<br>Malic acid	F2<br>Malic acid	F3<br>Malic acid	F4<br>Malic acid	F5<br>Tartaric acid	F6<br>Tartaric acid	F7<br>Tartaric acid	F8<br>Tartaric acid	F9<br>Fumaric acid	F10<br>Fumaric acid	F11<br>Fumaric acid	F12<br>Fumaric acid
G1<br>5-Fluorocytosine	G2<br>5-Fluorocytosine	G3<br>5-Fluorocytosine	G4<br>5-Fluorocytosine	G5<br>Palladium(II) Chloride	G6<br>Palladium(II) Chloride	G7<br>Palladium(II) Chloride	G8<br>Palladium(II) Chloride	G9<br>Ibuprofen	G10<br>Ibuprofen	G11<br>Ibuprofen	G12<br>Ibuprofen
H1<br>Chloroquine	H2<br>Chloroquine	H3<br>Chloroquine	H4<br>Chloroquine	H5<br>Cinnamic acid	H6<br>Cinnamic acid	H7<br>Cinnamic acid	H8<br>Cinnamic acid	H9<br>5-Fluorouracil	H10<br>5-Fluorouracil	H11<br>5-Fluorouracil	H12<br>5-Fluorouracil
