# Supplementary material for: Phenotype profiling of white-nose syndrome pathogen Pseudogymnoascus destructans and closely-related Pseudogymnoascus pannorum reveals metabolic differences underlying fungal lifestyles
Source: F1000Res. 2018 Jul 17;7:665. Originally published 2018 May 25. [Version 2] doi: 10.12688/f1000research.15067.2 (PMC6039956; doi:10.12688/f1000research.15067.2)
Supplement: Peer review history

# Peer review: Referee response for version 2

**DOI:** 10.5256/f1000research.17010.r36149

**Published:** 2018-08-02

**Authors:** Christine Salomon

**Affiliations:** 1 Center for Drug Design, University of Minnesota, Minneapolis, MN, USA

The authors have addressed my concerns and questions in their revision.

I have read this submission. I believe that I have an appropriate level of expertise to confirm that it is of an acceptable scientific standard.

# Peer review: Referee response for version 2

**DOI:** 10.5256/f1000research.17010.r36150

**Published:** 2018-08-01

**Authors:** Flavia Pinzari

**Affiliations:** 1 Council for Agricultural Research and Economics, Research Centre for Agriculture and Environment (CREA-AA), Rome, Italy

I think the answers and related changes to the text clarified most of the points.

I have read this submission. I believe that I have an appropriate level of expertise to confirm that it is of an acceptable scientific standard.

# Peer review: Referee response for version 1

**DOI:** 10.5256/f1000research.16406.r35120

**Published:** 2018-06-29

**Authors:** Flavia Pinzari

**Affiliations:** 1 Council for Agricultural Research and Economics, Research Centre for Agriculture and Environment (CREA-AA), Rome, Italy

The manuscript is focussed on a comparison between two *Pseudogymnoascus *fungal species, which belong to different species but have a partly overlapped ecological niche, based on the use of metabolic profiling with Biolog Phenotype Microarray commercial multiwell plates.

 These plates are used with redox dyes to evaluate substrate use. With fungi this colorimetric approach is very complicated, and as the authors actually did, the fungal growth in the wells is usually measured as a change in optical density.

 The interesting aspects and merits of this work are the following:

- This is a brilliant use of this technique, since the comparison between a pathogenic/parasitic species and a mainly saprophytic one can really highlight important clues on the nutritional requirements for the pathogenic organism to spread and develop.
- The two compared species *P. destructans *and the closely related species *P.pannorum are *truly interesting from different points of view. They live in caves, they live at low temperatures, they are very close but behave differently, they attack mammals, etc.

The main criticisms regard:

-  The description of the methods (very poor: it is difficult to understand the procedure followed both in data production and analysis), and the way data are presented.
- Figure 1 is really useless. Figure 3 is informative, but little or nothing is reported on data analysis in the methodological section.
- Apparently, the authors harvested the two fungi for inoculum preparation in two different moments (different sporification time: 3 to 5 weeks, it can be very different). They also chose two different incubation times for comparing the catabolism of the two fungi. These choices should be discussed and justified. The two fungi have different development times. This can be the main reason for the differences observed. A better description and motivation of the chosen approach would make the work stronger and clearer.
- Instead of single time-point comparison the authors could have used empirical models and regression splines that allow extrapolation of curve parameters of biological interest, namely, lag time, maximum rate of increase and maximum absorbance.  Curve integration and the resulting area under the curve condenses these three parameters into a single estimate that can also be used to compare kinetics across substrates and samples. Curve parameters offer the main advantage of being independent of incubation time, while also accounting for potential differential rates of colour development across substrates and plates. An example of this approach is given in the following work: Canfora et al (2017) ^1^, and theory is reported in the references therein.
- The authors tested only one strain for each species (I agree that PM plates are very expensive, but drawing a result using only one strains in the comparison is a limitation in case of intraspecific variability, even if they used two replicates)
- The lowest value on the heat map is an OD of “zero”. How was data scaling performed?
- What the red and green lines stand for in Figure 3? Legends are needed.
- References list is lacking of some important elements. One is the following: Atanasova L, Druzhinina IS ^2^.
- The authors reported that predicted enzymes are related to the number of carbon sources that can be utilized by the two fungal species however, a better definition of the kind of correlations observed between genotype and phenotype is needed to better understand this connection.

I have read this submission. I believe that I have an appropriate level of expertise to confirm that it is of an acceptable scientific standard, however I have significant reservations, as outlined above.

## References

[1] CanforaLAbu-SamraNTartanusMŁabanowskaBHBenedettiAPinzariFMalusàE: Co-inoculum of Beauveria brongniartii and B. bassiana shows in vitro different metabolic behaviour in comparison to single inoculums. *Sci Rep*.2017;7(1) : 10.1038/s41598-017-12700-0 13102 10.1038/s41598-017-12700-0 29026120PMC5638874

[2] AtanasovaLDruzhininaIS: Review: Global nutrient profiling by Phenotype MicroArrays: a tool complementing genomic and proteomic studies in conidial fungi. *J Zhejiang Univ Sci B*.2010;11(3) : 10.1631/jzus.B1000007 151-68 10.1631/jzus.B1000007 20205302PMC2833400

# Response

**Published:** 2018-07-11

**Authors:** Vishnu Chaturvedi

**Affiliations:** New York State Department of Health, USA

We appreciate the thoughtful comments of the reviewer.

**The main criticisms regard:**

**The description of the methods (very poor: it is difficult to understand the procedure followed both in data production and analysis), and the way data are presented.**

 We have added required details in the methods.

**Figure 1 is really useless. Figure 3 is informative, but little or nothing is reported on data analysis in the methodological section.**

 Figure 1 provided a bird’s-eye view of the scope of work and different trends noticed in this comparative study. We appreciate that someone knowledgeable about Biolog typing might find this information redundant and therefore, figure 1 was

 removed from the revised version. We expanded the legend for figure 3 (now figure 2).

**Apparently, the authors harvested the two fungi for inoculum preparation in two different moments (different sporification time: 3 to 5 weeks, it can be very different). They also chose two different incubation times for comparing the catabolism of the two fungi. These choices should be discussed and justified. The two fungi have different development times. This can be the main reason for the differences observed. A better description and motivation of the chosen approach would make the work stronger and clearer.**

 The reviewer’s criticism is valid similar to the reviewer 1. We have added details in the revised version to ally concern that “differences observed were due to different development times”.

**Instead of single time-point comparison the authors could have used empirical models and regression splines that allow extrapolation of curve parameters of biological interest, namely, lag time, maximum rate of increase and maximum absorbance.  Curve integration and the resulting area under the curve condenses these three parameters into a single estimate that can also be used to compare kinetics across substrates and samples. Curve parameters offer the main advantage of being independent of incubation time, while also accounting for potential differential rates of colour development across substrates and plates. An example of this approach is given in the following work: Canfora et al (2017) ^1^, and theory is reported in the references therein.**

 We have used the biolog phenotype plates as per the manufacturer’s instructions. There are many publications on Biolog profiling that use single end-point reading for trend analysis. The reviewer refers to a more elaborate setup, and we agree that studies similar to Canofora et al. (2017) provide more accurate and dynamic data for building predictable models. This reference was included in the bibliography and a sentence added in the revised text about the limitation of this qualitative study, and the scope for more dynamic data generation. We shall pursue future opportunities to conduct such a study with the two fungi.

**The authors tested only one strain for each species (I agree that PM plates are very expensive, but drawing a result using only one strains in the comparison is a limitation in case of intraspecific variability, even if they used two replicates)**

 We believe that a trend analysis is possible and meaningful with one well-defined strain representative from each species. This is also the basis for whole genome sequencing of most fungi.

**The lowest value on the heat map is an OD of “zero”. How was data scaling performed?**

 We have added more details to describe the heat map.

**What the red and green lines stand for in Figure 3? Legends are needed**

 We have modified the legends for the figure 6 (now figure 5). The red and green lines were deleted.

**References list is lacking of some important elements. One is the following: Atanasova L,Druzhinina IS ^2^.**

 We have included the article by Druzhinina et al.

**The authors reported that predicted enzymes are related to the number of carbon sources that can be utilized by the two fungal species however, a better definition of the kind of correlations observed between genotype and phenotype is needed to better understand this connection.**

 We modified the text to suggest that the trend analysis of Biolog data indicated correlation with the *in silico* analysis of genome sequencing data.

# Peer review: Referee response for version 1

**DOI:** 10.5256/f1000research.16406.r34574

**Published:** 2018-06-15

**Authors:** Christopher T Cornelison

**Affiliations:** 1 Division of Research and Advanced Studies, Kennesaw State University, Kennesaw, GA, USA

The manuscript, "Phenotype profiling of white-nose syndrome pathogen *Pseudogymnoascus destructans* and closely-related *Pseudogymnoascus pannorum *reveals metabolic differences underlying fungal lifestyles", describes the comparative analysis of metabolic profiling of 2 closely related fungal pathogens with vastly different hosts and virulence. The manuscript utilizes the well known BioLog system to accomplish the comparison. In general the the study is well designed and executed and the manuscript well written. The conclusions are not surprising considering previous publications regarding the genomics of Pd and its loss of carbon utilization related gene content. Accordingly the impact of the findings on the field are modest and the sophistication of the analysis is simplistic. Regardless the manuscript does support previous findings and although the methods are limited in scope they are sound and well vetted. Accordingly it is my recommendation that the manuscript is acceptable as it is.

I have read this submission. I believe that I have an appropriate level of expertise to confirm that it is of an acceptable scientific standard.

# Response

**Published:** 2018-07-11

**Authors:** Vishnu Chaturvedi

**Affiliations:** New York State Department of Health, USA

We appreciate very much the encouraging comments of the reviewer and the approval of the manuscript as it is.

# Peer review: Referee response for version 1

**DOI:** 10.5256/f1000research.16406.r34411

**Published:** 2018-06-07

**Authors:** Christine Salomon

**Affiliations:** 1 Center for Drug Design, University of Minnesota, Minneapolis, MN, USA

This paper describes a relatively straightforward study focused on comparing the nutrient utilization capacity of the fungal bat pathogen *Pseudogymnoascus destructans* versus the closely related species *P. pannorum *using the well established Biolog phenotype system. In general, the results suggest that *P. pannorum* can more readily utilize most carbon and nitrogen sources compared to *P. destructans* under the experimental conditions tested (15 deg C, and 7 and 10 days, respectively). The bat pathogen was also more sensitive to pH extremes and less tolerant to high salt.   The authors conclude that these results validate their previous whole genome studies which compared the predicted protein numbers between these two species. In general, the results also support previous metabolic capacity studies of *P. destructans* and other non-pathogenic *Pseudogymnoascus* species.

 There are some important questions that should be addressed, and additional details that would improve this manuscript:

- How was the incubation time of 10 and 7 days for each species determined? Presumably by comparison of equivalent growth in the control wells, but this detail should be provided.  If this time is increased, does the utilization capacity of *P. destructans* eventually catch up?
- More details should be provided about the cut-off value determination for growth versus no growth.  Also, there should be some analysis of the range of results, versus simply using the average of the two readings. For example, what was the standard deviation for replicates?
- It would be helpful to have a map of the nutrient sources, xenobiotics, etc.  in the supplementary data to accompany the OD data (the numerical data alone is impossible to interpret without any other identifying information)--presumably Biolog provides this as a document.
- It’s not clear how the heat map values were generated, if the starting spore inoculum had an OD of 0.2.  (since the lowest value on the heat map is “0” OD). Presumably, if no growth occurred under a given condition, it would remain at the starting OD? Also, is 1.0 the highest OD obtained or was the data scaled to 0-1.0?
- The heat map figure for the “nutrient supplements” is missing (also, I’m not sure what compounds this category encompasses, so some information about this would also be helpful, perhaps even just referring to the plate map in the supplementary data if that is added).
- For Figure 6, the solid and dotted green and red lines seem to indicate relative growth, but the numerical cutoffs should be provided in the methods (or figure legend). For example, the growth in the well for 2% NaCl looks (labeled with a solid green line above) looks similar to the well for pH 9 with a dotted green line, but presumably are numerically different.  
- Part of the justification for doing this work is stated as confirming the in silico gene predictions (from a previous publication by the authors). However, it’s not entirely clear that the just comparing the overall numbers of predicted proteins is actually correlated to the overall number of nutrient sources that can be utilized. This seems likely to be true, but the two studies don’t necessarily test/confirm this connection.  The reference cited is a short report on the overall sequencing of the *P. destructans* and *P. pannorum* genomes, and prediction of encoded proteins, but no significant functional analysis.  It might be more relevant to include references that include more functional data on metabolic and enzymatic capacities.  

Overall, this work adds important information about the competitive ability and metabolic specificity of *P. destructans* and could provide additional insight into fungal life history strategies and potential ways to control or mitigate white nose syndrome in bats. Some additional details (highlighted above) would provide critical information that would allow others to replicate or expand on this work.

I have read this submission. I believe that I have an appropriate level of expertise to confirm that it is of an acceptable scientific standard, however I have significant reservations, as outlined above.

# Response

**Published:** 2018-07-11

**Authors:** Vishnu Chaturvedi

**Affiliations:** New York State Department of Health, USA

Thanks very much for your insightful comments on the manuscript. We have modified the manuscript given your suggestions.

**How was the incubation time of 10 and 7 days for each species determined? Presumably by comparison of equivalent growth in the control wells, but this detail should be provided.  If this time is increased, does the utilization capacity of *P. destructans* eventually catch up?**

 In preliminary experiments, both fungi grew at different growth rates and comparable growth was observed after day 7 for *P. pannorum* and day 10 for *P. destructans* (details not shown). Further incubation of the plates beyond the observation period did not change the observed growth pattern.

**More details should be provided about the cut-off value determination for growth versus no growth.  Also, there should be some analysis of the range of results, versus simply using the average of the two readings. For example, what was the standard deviation for replicates?**

 The PM plates were inoculated per Biolog protocol and incubated at 15° C. The presence or absence of growth was measured by OD _600_ on day 7 for *P. pannorum* and day 10 for *P. destructans*. The negative control wells were weakly growth positive for both *P. destructans* and *P. pannorum*. The observation was also reported for the Biolog PM plates in another study (Nai C, et al. Fungal Genet Biol. 2013; 56:54-66). Therefore, the corresponding negative control well reading from each experiment were averaged together and used to normalize the OD values averages for each test compound. For the heat map visualization, the negative control reading was assigned a score 0.0 and the positive growth scored on a 0.0 -1.0 scale. The phenotypic assay was repeated once. The limited dataset precluded any quantitative statistical analyses.

**It would be helpful to have a map of the nutrient sources, xenobiotics, etc.  in the supplementary data to accompany the OD data (the numerical data alone is impossible to interpret without any other identifying information)--presumably Biolog provides this as a document**.

 The Biolog maps for all test compound are now included as a supplementary file. 

**It’s not clear how the heat map values were generated, if the starting spore inoculum had an OD of 0.2.  (since the lowest value on the heat map is “0” OD). Presumably, if no growth occurred under a given condition, it would remain at the starting OD? Also, is 1.0 the highest OD obtained or was the data scaled to 0-1.0?**

 Please see details provided earlier about the heat map visualization.

**The heat map figure for the “nutrient supplements” is missing (also, I’m not sure what compounds this category encompasses, so some information about this would also be helpful, perhaps even just referring to the plate map in the supplementary data if that is added).**

 The nutrient map from Biolog was uploaded as a supplementary file. 

**For Figure 6, the solid and dotted green and red lines seem to indicate relative growth, but the numerical cutoffs should be provided in the methods (or figure legend). For example, the growth in the well for 2% NaCl looks (labeled with a solid green line above) looks similar to the well for pH 9 with a dotted green line, but presumably are numerically different.**

 The legend for figure 6 (now figure 5) was modified for clarity; the summary numerical cutoff values were included in the revised text.

**Part of the justification for doing this work is stated as confirming the in silico gene predictions (from a previous publication by the authors). However, it’s not entirely clear that the just comparing the overall numbers of predicted proteins is actually correlated to the overall number of nutrient sources that can be utilized. This seems likely to be true, but the two studies don’t necessarily test/confirm this connection.  The reference cited is a short report on the overall sequencing of the *P. destructans* and *P. pannorum* genomes, and prediction of encoded proteins, but no significant functional analysis.  It might be more relevant to include references that include more functional data on metabolic and enzymatic capacities.**  

 Instead of ‘confirm,’ we switched to ‘assess correlations with *in silico* gene predictions.’ The two studies provide initial information about the genomes and metabolic pathways. The findings indicate a trend towards unique genomic and phenomic attributes in two psychrophilic fungi with different lifestyles. We agree with the reviewer that there is need to carry out more detailed functional analysis to identify unique genes, enzymes, and other proteins that differentiate these two psychrophiles. Such studies, necessitating considerable resources, are planned for the future.
